# The Search of miRNA Related to Invasive Growth of Nonfunctioning Gonadotropic Pituitary Tumors

**DOI:** 10.1155/2020/3730657

**Published:** 2020-12-05

**Authors:** Joanna Boresowicz, Paulina Kober, Natalia Rusetska, Maria Maksymowicz, Agnieszka Paziewska, Michalina Dąbrowska, Natalia Zeber-Lubecka, Jacek Kunicki, Wiesław Bonicki, Jerzy Ostrowski, Janusz A. Siedlecki, Mateusz Bujko

**Affiliations:** ^1^Department of Molecular and Translational Oncology, Maria Sklodowska-Curie National Research Institute of Oncology, Warsaw, Poland; ^2^Department of Pathology and Laboratory Diagnostics, Maria Sklodowska-Curie National Research Institute of Oncology, Warsaw, Poland; ^3^Department of Genetics, Maria Sklodowska-Curie National Research Institute of Oncology, Warsaw, Poland; ^4^Department of Gastroenterology, Hepatology and Clinical Oncology, Medical Center for Postgraduate Education, Warsaw, Poland; ^5^Department of Neurosurgery, Maria Sklodowska-Curie National Research Institute of Oncology, Warsaw, Poland

## Abstract

**Purpose:**

Nonfunctioning gonadotropic pituitary neuroendocrine tumors (PitNETs) are among the most frequent neoplasms of pituitary gland. Although PitNETs are commonly considered benign, a notable part of patients suffer from tumor recurrence after treatment. Invasive growth of pituitary tumor is among the most important prognostic factors. Since molecular features of invasiveness are of potential clinical usefulness, this study was aimed to verify whether invasive and noninvasive nonfunctioning gonadotropic PitNETs differ in the miRNA expression profile and whether the differences could provide a possible molecular classifier.

**Methods:**

miRNA profiles were determined in 20 patients (11 invasive and 9 noninvasive tumors) using next-generation sequencing. The expression of selected miRNAs was assessed in the independent cohort of 80 patients with qRT-PCR.

**Results:**

When miRNA profiles of invasive and noninvasive tumors were compared, 29 miRNAs were found differentially expressed. Hsa-miR-184, hsa-miR-181a-2-3p, hsa-miR-93-3p, hsa-miR-574-5p, hsa-miR-185-5p, and hsa-miR-3200-5p showed a potential clinical value according to ROC curve analysis. Unfortunately, differential expression of only hsa-miR-185-5p was confirmed in the validation cohort, with AUG at 0.654.

**Conclusion:**

Differences in miRNAs expression profiles in invasive and noninvasive gonadotropic PitNETs are slight and the level of miRNA expression seems not to be applicable as useful classifier of tumor invasiveness.

## 1. Introduction

Pituitary neuroendocrine tumors (PitNETs) are the third most common intracranial neoplasms in adults, accounting for approximately 16.5% of all tumors in this location [1]. They are classified into subtypes originating from the particular functional type of pituitary cells and are characterized by overproduction of specific hormones [2]. Approximately, 33% of PitNETs are clinically nonfunctioning tumors, which do not cause any specific endocrinological symptoms and the bulk of nonfunctioning PitNETs are those derived from gonadotroph pituitary cells [3].

Nonfunctioning PitNETs are considered benign neoplasms; however, a large proportion of them are characterized by the invasive growth that is manifested by infiltration of adjacent structures by the tumor. Invasive growth has important clinical implications because it hampers complete tumor resection, which is a basic treatment of nonfunctioning gonadotroph tumors and results in tumor recurrence [2–4]. It is also one of the most important prognostic features for patients suffering from PitNETs [4].

MicroRNAs (miRNAs) are small noncoding RNA particles that play a pivotal role in posttranscriptional gene silencing. They act mainly as gene silencers through interaction with mRNA particles containing fully or partially complementary sequence [5]. MicroRNAs were proven to regulate up to 30% of all proteins encoded in the human genome [6]. Abnormal expression of miRNAs contributes to pathogenesis of various human neoplasms [7]. Dysregulation of pathways of miRNA biogenesis and action may impact all the cellular processes relevant in cancer development [7]. It became clear that they play a role in pathogenesis of pituitary tumors as well [8, 9].

A number of miRNAs were shown to be differentially expressed in nonfunctioning pituitary tumors compared with normal pituitary [8]. These aberrant miRNA levels cause the change of the expression level of a known pituitary tumorigenesis driver genes as those encoding for HMGA proteins [10], cyclin B1 [11], or CDC25 [12] and lead to deregulation of the important pathways including PI3K/AKT [13] and TGFbeta signaling [14].

The impaired miRNA expression contributes to invasiveness and metastasis of human cancers [15]. miRNAs were also shown to play a role in the acquisition of the ability to infiltrate adjacent tissues in different subtypes of pituitary tumors [16].

In this study, we made an attempt to identify miRNAs with expression related to invasive growth of nonfunctioning gonadotroph PitNETs. For this purpose, the whole miRNA expression profiles of invasive and noninvasive tumors were compared, and subsequently, the expression of selected miRNAs was determined in an independent patients' cohort.

## 2. Methods

### 2.1. Patients and Samples

Tissue samples from 100 patients were collected during transsphenoidal surgery and immediately frozen in liquid nitrogen for storage at −80°C. Part of each tumor was assessed histopathologically. Histopathological assessment included both ultrastructural investigation and immunohistochemistry according to WHO 2004 criteria, which were applicable at the time of sample collection [17]. Tumor invasion was evaluated with preoperative MRI, and main directions of invasive growth were assessed. Cavernous sinus invasion was defined by extension of adenoma beyond the line corresponding to the lateral tangents of the intracavernous carotid artery (Grades 3 and 4) as defined by Micko et al. [18, 19]. Infrasellar direction of invasive growth was considered if bone and dura of the sellar floor, sphenoid sinus, and clivus were invaded. The suprasellar expansion was considered as invasive only if leptomeningeal infiltration was observed. Invasiveness of pituitary adenomas was taken into account only if it was intraoperatively confirmed by the surgeon with endoscopic inspection of infiltrated areas and/or by histology.

The study included gonadotropic tumors that represent the most common histopathological subtype of nonfunctioning PitNETs. Five patients with null cell adenomas were also enrolled. These five samples were immunonegative for all hormones but clearly showed features of gonadotropinomas in ultrastructural examination. Patient profiles are presented in Table 1, and detailed patients' clinical data are presented in Supplementary Table S1.

Total RNA was isolated using the MirVana miRNA Isolation Kit (Thermo Fisher Scientific) and rotor stator homogenizer Omni Tissue Master (Omni International). The RNA quality was assessed spectrophotometrically using the NanoDrop 2000 (Thermo Fisher Scientific). Isolated RNA was stored at −80°C until analyses.

### 2.2. Assessment of miRNA Expression Profile with Next-Generation Sequencing (NGS)

The quality of small RNA fractions was assessed using Agilent 2100 Bioanalyzer with Small RNA Kit Chip (Agilent) and measured with Qubit RNA HS Assay Kits (Thermo Fisher Scientific). One *μ*g of total RNA was used for sequencing library construction with an Ion Total RNA-Seq Kit v2 (Thermo Fisher Scientific), according to the manufacturer's protocol. Ion Xpress™ RNA-Seq Barcode Kit was used for hybridization and ligation of RNA adapters that allows for multiplexed sequencing. RNA reverse transcription and subsequent cDNA purification and library size selection were performed using Nucleic Acid Binding Beads. cDNA was PCR-amplified, followed by DNA purification and size selection. The amount and size distribution of the amplified DNA was determined using Bioanalyzer 2100 using a High Sensitivity DNA Kit (Agilent). The length of miRNA ligation products in barcoded libraries ranged between 94 and114 bp. Template preparation for clonal amplification of up to four miRNA libraries at a concentration of 18 pM and loading of the PI chip were performed using Ion Chef Instrument, with Ion PI™ Hi-Q™ Chef Kit (Thermo Fisher Scientific). Ion Proton Sequencer (Thermo Fisher Scientific) was used for sequencing. Unmapped bam files were converted into fastq files with a bamToFastq script from bedtools. Read mapping to known human miRNAs (according to miRBase v.22) and reads quantification were performed using miRDeep2.14. Data normalization and differential expression analysis were performed using DESeq2. Filtration for low-expression miRNAs and miRNA genes with less than five sequencing reads in at least half of the samples were excluded. Fold change of expression (FC), calculated as read counts ratio of invasive/noninvasive NFPAs, was used as a measure of expression difference between the groups of samples. Differentially expressed miRNAs were defined as those which met criterion of *p*-value <0.05.

### 2.3. qRT-PCR-Based miRNA Expression Assessment

miRCURY LNA™ miRNA PCR System (Qiagen) was used for measurement of miRNA expression levels. Fifty nanograms of total RNA was reverse-transcribed with miRCURY LNA™ Universal RT miRNA PCR and cDNA synthesis kit (Exiqon). cDNA was diluted x25. qPCR reactions were performed in 384-well format using 7900HT PCR system (Applied Biosystems). qPCR reaction of 5 *μ*l volume contained 2 *μ*l of diluted cDNA, 1x SYBR Green Master Mix (Exiqon), and miRNA LNA PCR primer set (Exiqon). The following predesigned miRCURY LNA™ primer assays were used: YP00204601 (hsa-miR184), YP00204142 (hsa-miR181a-2-3p), YP00204470 (hsa-miR-93-3p), YP02116206 (hsa-miR-574-5p), YP00206037 (hsa-miR-185-5p), YP02109903 (hsa-miR-3200-5p), YP00203906 (5s RNA), YP00203907 (U6 RNA), YP00203902 (SNORD44), and YP00203905 (SNORA66) (Qiagen). All qRT-PCR measurements were performed in technical triplicate. The 2^−ΔCT^ method was used for calculation of relative expression level. Reference genes were selected upon the evaluation of expression stability using RefFinder software [20].

### 2.4. Statistical Analysis

Quantitative continuous variables were analyzed by a two-sided Mann–Whitney *U* test. ROC curve analyses were applied for testing potential diagnostic value. The two-sided Fisher's exact test was used for analyzing proportions. Data were analyzed and visualized using GraphPad Prism (GraphPad Software). Heatmap graphs and hierarchical clustering were done with Cluster 3.0 and TreeView 1.6 software (Stanford University School of Medicine, Stanford, CA, USA). Complete linkage clustering with Euclidean distance as similarity metrics were used.

## 3. Results

### 3.1. Identification of miRNAs with Differential Expression in Invasive and Noninvasive NFPAs

Twenty gonadotropic PitNET samples, including 11 invasive and 9 noninvasive tumors, were subjected to miRNA expression analysis with next-generation sequencing of small RNA using semiconductor sequencing. Sequencing of small RNA libraries generated an average of 2,497,367 reads per sample, which were mapped to the human genome (hg19) and used for quantification of expression levels of known miRNAs. Sequencing reads were annotated to 1,528 miRNAs, and after filtering out those with low expression, the measurements of 405 miRNAs were used in the analyses.

**Figure 1:**
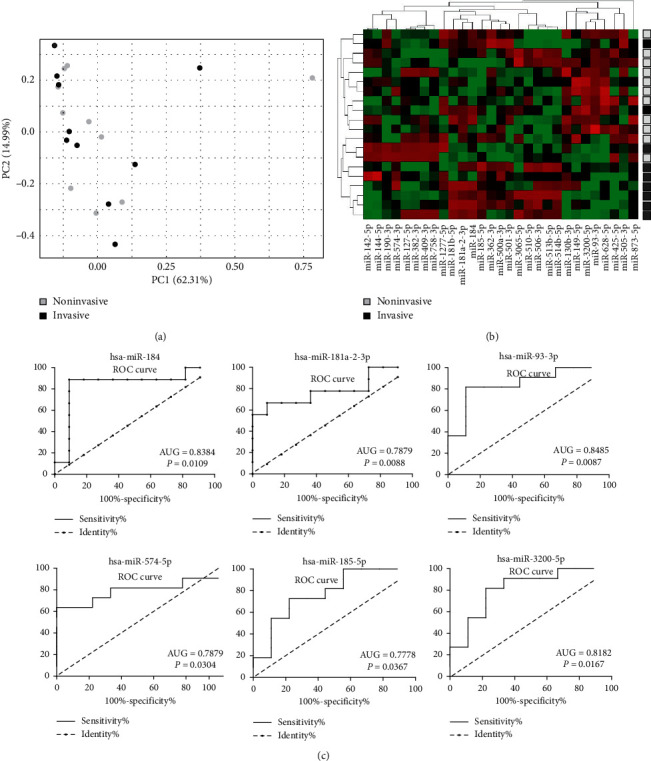
Comparison of miRNA expression in invasive and noninvasive gonadotropic PitNETs with next-generation sequencing. (a) Principal component analysis; (b) heatmap and hierarchical clustering using normalized read counts value for differentially expressed miRNAs; (c) ROC curve analysis for six miRNAs considered as potential markers of invasive growth.

The principal component analysis (PCA) (Figure 1(a)) indicates that the potential differences of miRNA expression in invasive and noninvasive PitNETs are small, and no clear distinction between tumors with invasive and noninvasive growth was observed.

When miRNA expression profiles of invasive and noninvasive tumors were compared to identify miRNA with different expression levels, 29 miRNAs met the criterion of *p*-value <0.05. Only seven of them met *p*-value <0.01, and none met adjusted *p*-value <0.05. Expression of 29 differentially expressed miRNAs is visualized with heatmap in Figure 1(b) and the results are presented in Table S2.

When hierarchical clustering was applied for clustering the samples using normalized read counts for 29 differentially expressed miRNAs, two main clusters were observed with different proportions of invasive and noninvasive PitNETs (*p*=0.0216); however, an explicit classification into invasive and noninvasive tumors was not found (Figure 1(b)).

ROC curve analysis was applied for all differentially expressed miRNAs to determine which of them may potentially serve as classifiers of tumor invasiveness status. Six miRNAs showed AUG significantly higher than 0.5, including hsa-miR-184 AUG = 0,8384 (95% CI 0.6299 to 1.047, *p*=0,0109); hsa-miR-181a-2-3p AUG = 0,7879 (95% CI 0.5704 to 1.005, *p*=0.0304); hsa-miR-93-3p AUG = 0.8485 (95% CI 0.6716 to 1.025, *p* = 0.0088); hsa-miR-574-5p AUG = 0,7879 (95% CI 0.5736 to 1.002, *p*=0.0304); hsa-miR-185-5p AUG = 0.7778 (95% CI 0.5656 to 0.9900, *p*=0.03673); hsa-miR-3200-5p AUG = 0.8182 (95% CI 0.6237 to 1.013, *p*=0.01674). ROC curves are presented in Figure 1(c), while the detailed results are presented in Supplementary Table 2. These six miRNAs, considered as potential markers of invasiveness, were selected for qPCR validation analysis on the independent patients' cohort.

### 3.2. Validation

The expression levels of the selected six miRNAs were determined with qRT-PCR in 20 PitNET samples that were subjected to sequencing-based analysis and in 80 additional tumor tissues from 80 patients from independent validation cohort.

Reference genes for normalizing qRT-PCR data were selected based on testing the stability of expression of four small RNAs: 5S RNA, U6 RNA, *SNORD44*, and *SNORA66.* Expression of these RNAs was assessed in PiTNET samples, and their usefulness as reference genes was determined using RefFinder software [20]. Since expression of *SNORA66* was below qPCR detection level in the large proportion of samples, it was not included in the analysis of expression stability. Based on the examination of candidate references (shown in Supplementary Figure S1), the geometric mean of Ct values from two references *SNORD44* and 5S RNA was used to normalize expression data.

**Figure 2:**
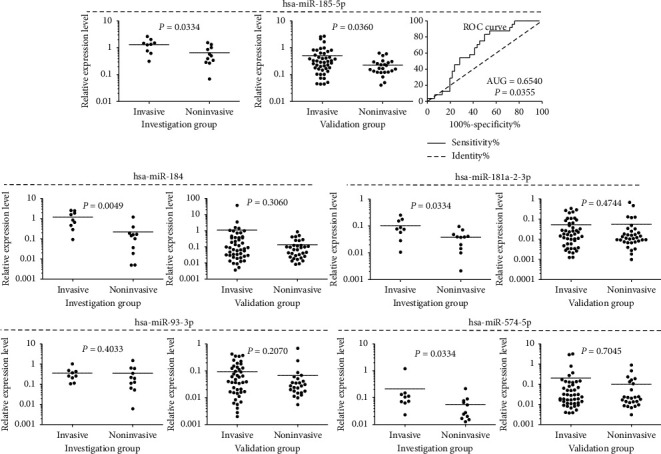
Comparison of invasive and noninvasive gonadotropic PitNETs in terms of expression level of selected miRNAs measured with qRT-PCR assay. Analyses of discovery and validation groups are presented in separate graphs. Mean values are horizontal lines.

qRT-PCR results confirmed different expression of hsa-miR-184, hsa-miR-181a-2-3p, hsa-miR-574-5p, and hsa-miR-185-5p4 in discovery group of 20 patients that was observed in the analysis of NGS results, as shown in Figure 2. Differential expression of hsa-miR-93-3p observed in the sequencing analysis was not confirmed by qRT-PCR assay. qRT-PCR results of hsa-miR-3200-5p expression assessment were excluded from the analysis, because most of the measurements (over 75%) were PCR nondetects. This made a reliable comparison impossible.

hsa-miR-185-5p was the only miRNA with differential expression level in the PitNET samples of validation cohort stratified according to invasiveness status (FC = 2.2, *p*=0.0360 ). Unfortunately, the results of ROC curve analysis with AUG value of 0.654 indicate that this miRNA is not a useful classifier of invasiveness status. No significant difference in the expression of hsa-miR-184, hsa-miR-181a-2-3p, hsa-miR-93-3p, or hsa-miR-574-5p was observed between invasive and noninvasive tumors. The results of qRT-PCR-based analysis are presented in Figure 2.

## 4. Discussion

The majority of nonfunctioning gonadotropic PitNETs are benign and slow-growing tumors, and patients achieve a long-term remission after tumor resection, which is the basic method of treatment. Unfortunately, some tumors are characterized by a more aggressive growth and represent an important clinical problem. Invasive growth of pituitary tumor is considered one of the main causes of tumor recurrence, and it is a commonly approved prognostic factor of increased risk of recurrence after surgery [21].

The molecular background of invasive growth of pituitary tumors is still unclear; however, it is believed that specific biological mechanisms contribute to their more aggressive growth and infiltrating nature [16]. The possible biological determinants of invasive growth are of potential clinical usefulness as they could serve as additional prognostic factors. Patients with a well-defined high risk of recurrence could benefit from more intensive adjuvant treatment or more frequent follow-up visits. For this reason, an effort is being made to identify molecular features that are related to invasive growth by the search of different layers of molecular profiling: genetic, transcriptomic, epigenetic, and proteomic [16].

miRNAs play a role in posttranscriptional regulation of gene expression and protein translation. Since impaired miRNA expression can affect cellular phenotype, it also may potentially trigger the ability of invasive growth in cancer cells [15]. This is supported by increasing number of reports showing the role of particular miRNAs in aggressive growth of different subtypes of pituitary tumors [8, 9, 16].

In our study, we determined the whole profile of miRNA expression in nonfunctioning gonadotropic pituitary tumors and tried to verify the hypothesis that invasive and noninvasive PitNETs differ in expression of miRNAs. The analysis of whole miRNA expression profile involved 20 patients with a particular subtype of pituitary tumors, and due to this relatively small number of patients, we used a relaxed statistical criterion of unadjusted *p* < 0.05 for differentially expressed miRNAs. We believe this could be applied since additional validation on independent patients cohort was planned for this study. With this criterion, 29 miRNAs could be identified as differentially expressed, but classification of the discovery set of PitNET samples based on these miRNAs did not allow for the unequivocal distinction between invasive and noninvasive tumors. These results, along with the basic overall PCA data, allow for the conclusion that tumors stratified according to invasiveness status hardly differ in the miRNA expression profile.

A few reports showing the role of miRNA in invasive growth of different subtypes of pituitary tumors were published [16]. Since recent data indicated a notable difference in miRNA profile between PitNETs derived from different functional subtypes of pituitary cells [22] when comparing our results with those previously published we focused selectively on studies analyzing non-functioning PitNETs.

A research most comparable to our study has been published by Wu S et al., who compared miRNA profiles in invasive and noninvasive nonfunctioning pituitary tumors with microarrays [23]. In the analysis of 12 tumor samples, the authors found 10 differentially expressed miRNAs that met the criterion of unadjusted *p* < 0.05 and fold change >2. None of these miRNAs has been identified in our data, which may be partially explained by the fact that the authors included combined gonadotropinomas and silent ACTH and GH-producing tumors in the study group.

In the studies focused on a priori selected particular miRNAs in nonfunctioning pituitary tumors, differential expression in invasive and noninvasive PitNETs was observed in hsa-miR-376B-3p [24] as well as in hsa-miR-137, hsa-miR-374a-5p, and hsa-miR-374b-5p [25]. These results have not been confirmed by our results, since none of these miRNAs was found as differentially expressed in tumors stratified according to invasive growth status.

This inconsistency of data from different investigations is in line with a more general, apparent low reproducibility of the results of the research on invasion-related molecules. The results of most studies, including those with high-throughput methods, are not consistent with the other ones, as highlighted in recently published review articles [4, 26]. This may reflect different methodological problems including patients' numbers, heterogeneity of patient groups, and differences between laboratory techniques. The use of different analytical platforms affects the results of miRNA profiling as clearly shown in pituitary tumors [27], which also may partially explain the lack of common invasiveness-related miRNA in our data and those previously obtained by Wu et al. with microarrays [23].

Additionally, the molecular background of invasive growth of pituitary tumors seems to be complex. Many different molecular changes, different regulatory layers, and signaling pathways play a role in acquisition of invasive phenotype by the cells [16]. Comparing the results of different studies is also difficult due to slightly different criteria for determining the status of invasive growth used by various researchers [28].

Since classifiers of tumors invasiveness status are of potential clinical usefulness, we applied ROC curve analysis for 29 miRNAs differentially expressed in invasive and noninvasive tumors. This allowed for identification of six miRNAs: hsa-miR-184, hsa-miR-181a-2-3p, hsa-miR-93-3p, hsa-miR-574-5p, hsa-miR-185-5p, and hsa-miR-3200-5p that could potentially serve as biomarkers of invasive growth potential.

Little is known about the role of these miRNAs in pituitary tumors. Only one of them, hsa-miR-93, was previously described as related to invasive growth status of PitNETs. Its decreased expression was observed in invasive nonfunctioning tumors [29], according to our NGS-based results. On the contrary, in corticotropic PitNETs, its expression was found higher in invasive tumors than in noninvasive ones. Hsa-miR-184 was found upregulated in GH-producing pituitary tumors compared with normal pituitary [22], while hsa-miR-185-5p is overexpressed in GHomas not responding to SSTR treatment, and its high expression corresponds to high proliferation and inhibits the apoptosis of GH3 pituitary adenoma cells [30].

When focusing on the more general role of these miRNAs in human tumors, contradictory results can be found in the literature indicating their role as both oncogenic and oncosuppressive depending on tumor type. Hsa-miR-184 seems to be a suppressive miRNA in some tumors like glioma and nasopharyngeal carcinoma [31, 32], but it is overexpressed in adrenocortical carcinoma and renal carcinoma, where the expression level is stepwise growing along with tumor stage. Hsa-miR-185-5p enhances proliferation of pituitary cells [30], but it inhibits invasion and migration of colon cancer and hepatocellular carcinoma [33, 34]. Hsa-miR-181a-2-3p was reported as overexpressed in head and neck cancer and adenoid cystic carcinoma [35, 36], while it is downregulated in papillary thyroid carcinoma [37]. Upregulation of hsa-miR-3200 inhibits migration and invasion of gastric cancer cells, [38] and conversely, it promotes the invasion of osteosarcoma cells [39]. Similarly, hsa-miR-93-3p [40] and hsa-miR-574-5p [41, 42] may also play both oncogenic and suppressive roles.

Validation of the usefulness of six putative miRNA classifiers of invasive growth was performed by PCR-based analysis of expression of selected miRNAs in the independent validation cohort of 80 patients that were classified with the same criteria as discovery group patients to exclude differences in defining tumor invasive growth. qRT-PCR analyses were done on both discovery and validation cohorts to exclude the impact of different methods on validation procedure.

Significant difference between invasive and noninvasive tumors was observed only for one of the selected miRNAs, that is, hsa-miR-185-5p. Unfortunately, sensitivity and specificity analysis for this miRNA used as a classifier of invasive growth status did not confirm its usefulness as an effective marker.

qRT-PCR results of hsa-miR-184, hsa-miR-181a-2-3p, and hsa-miR-574-5p confirmed the difference of the tumors stratified according to the status of invasive growth in the discovery group, as identified with NGS that indicates the accordance of both analytical methods, but no significant difference was observed in validation cohort. Assessment of hsa-miR-93-3p expression with qRT-PCR assay did not confirm the difference between invasive and noninvasive PitNETs in neither discovery nor validation cohort. Thus, it did not confirm the previously reported downregulation of this miRNA in invasive nonfunctioning tumors [29].

We conclude that our results suggest the lack of universal miRNA profile of the tumor that determines invasive growth. It does not imply that expression of particular miRNA does not play a role in the aggressive phenotype of nonfunctioning pituitary tumors, as shown by some previous functional experiments [24, 25, 43]. We would rather assume that different mechanisms and various molecular changes may contribute to the acquisition of ability of infiltrative growth.

## 5. Conclusion

Differences in the levels of miRNAs between invasive and noninvasive nonfunctioning gonadotropic PitNETs are very slight, and tumor miRNA expression seems not to be a source of a promising classifier of invasiveness and potential biomarker.

## Figures and Tables

**Table 1:** Patients' characteristics.

	Discovery group	Validation group
**NFPA patients (number of patients)**	20	80

Age (years)		
Range	36–73	34–80
Median	58	63

Sex (number of patients)		
Male	17	44
Female	3	36

Histopathology (number of patients)		
Gonadotroph PitNET	20	75
Null-cell/gonadotroph PitNET	0	5

Clinical classification (number of patients)		
Invasive PitNET	11	47
Noninvasive PitNET	9	33

## Data Availability

Data of next-generation sequencing of miRNA in invasive and noninvasive gonadotropic PitNETs have been deposited in Gene Expression Omnibus database.

## Supplementary Materials

Supplementary Materials Table S1: patient characteristics in detail. Table S2: the results of comparison of miRNA expression in invasive and noninvasive gondotropic PitNETs. Figure S1: validation of selected small RNA molecules as the reference for normalization for QRT–PCR data for assessment of miRNA level in gondotroph pituitary neuroendocrine tumors.
