# Supplementary material for: The Search of miRNA Related to Invasive Growth of Nonfunctioning Gonadotropic Pituitary Tumors
Source: Int J Endocrinol. 2020 Dec 5;2020:3730657. doi: 10.1155/2020/3730657 (PMC7737439; doi:10.1155/2020/3730657)
Supplement: Supplementary Materials — Table S1: patient characteristics in detail. Table S2: the results of comparison of miRNA expression in invasive and noninvasive gondotropic PitNETs. Figure S1: validation of selected small RNA molecules as the reference for normalization for QRT–PCR data for assessment of miRNA level in gondotroph pituitary neuroendocrine tumors. [file 3730657.f1.zip › 3730657.f1/Supplementary description - 3730657.docx]

Supplementary description

Table S1. Patient characteristics in details.

Table S2. The results of comparison of miRNA expression in invasive and noninvasive gondotropic PitNETs.

Figure 1. Validation of selected small RNA molecules as the reference for normalization for QRT – PCR data for assessment of miRNA level in gondotroph pituitary neuroendocrine tumours
